# Proposed Definitions of Antibody-Mediated Rejection for Use as a Clinical Trial Endpoint in Kidney Transplantation

**DOI:** 10.3389/ti.2022.10140

**Published:** 2022-05-20

**Authors:** Candice Roufosse, Jan Ulrich Becker, Marion Rabant, Daniel Seron, Maria Irene Bellini, Georg A. Böhmig, Klemens Budde, Fritz Diekmann, Denis Glotz, Luuk Hilbrands, Alexandre Loupy, Rainer Oberbauer, Liset Pengel, Stefan Schneeberger, Maarten Naesens

**Affiliations:** ^1^ Department of Immunology and Inflammation, Centre for Inflammatory Disease, Imperial College London, London, United Kingdom; ^2^ Institute of Pathology, University Hospital Cologne, Cologne, Germany; ^3^ Department of Pathology, Hôpital Necker-Enfants Malades, Paris, France; ^4^ Department of Nephrology and Kidney Transplantation, Vall d’Hebrón University Hospital, Barcelona, Spain; ^5^ Department of Surgical Sciences, Sapienza University of Rome, Rome, Italy; ^6^ Division of Nephrology and Dialysis, Department of Internal Medicine, Medical University of Vienna, Vienna, Austria; ^7^ Department of Nephrology and Medical Intensive Care, Charité Universitätsmedizin Berlin, Berlin, Germany; ^8^ Department of Nephrology and Kidney Transplantation, Hospital Clinic Barcelona, Barcelona, Spain; ^9^ Paris Translational Research Center for Organ Transplantation, Hôpital Saint Louis, Paris, France; ^10^ Department of Nephrology, Radboud University Medical Center, Nijmegen, Netherlands; ^11^ Paris Translational Research Center for Organ Transplantation, Hôpital Necker, Paris, France; ^12^ Centre for Evidence in Transplantation, University of Oxford, Oxford, United Kingdom; ^13^ Department of General, Transplant and Thoracic Surgery, Medical University of Innsbruck, Innsbruck, Austria; ^14^ Department of Microbiology, Immunology and Transplantation, KU Leuven, Leuven, Belgium

**Keywords:** kidney transplantation, outcomes, biopsy, histology, antibody-mediated rejection, EMA guideline

## Abstract

Antibody-mediated rejection (AMR) is caused by antibodies that recognize donor human leukocyte antigen (HLA) or other targets. As knowledge of AMR pathophysiology has increased, a combination of factors is necessary to confirm the diagnosis and phenotype. However, frequent modifications to the AMR definition have made it difficult to compare data and evaluate associations between AMR and graft outcome. The present paper was developed following a Broad Scientific Advice request from the European Society for Organ Transplantation (ESOT) to the European Medicines Agency (EMA), which explored whether updating guidelines on clinical trial endpoints would encourage innovations in kidney transplantation research. ESOT considers that an AMR diagnosis must be based on a combination of histopathological factors and presence of donor-specific HLA antibodies in the recipient. Evidence for associations between individual features of AMR and impaired graft outcome is noted for microvascular inflammation scores ≥2 and glomerular basement membrane splitting of >10% of the entire tuft in the most severely affected glomerulus. Together, these should form the basis for AMR-related endpoints in clinical trials of kidney transplantation, although modifications and restrictions to the Banff diagnostic definition of AMR are proposed for this purpose. The EMA provided recommendations based on this Broad Scientific Advice request in December 2020; further discussion, and consensus on the restricted definition of the AMR endpoint, is required.

## What is Antibody-Mediated Rejection?

Although biopsy-proven acute rejection (BPAR) remains widely used as a primary efficacy variable in the clinical trial setting (1), it is a non-specific term. Despite often considered equivalent to acute T cell-mediated rejection (aTCMR), BPAR likely also includes unrecognized cases of antibody-mediated injury, especially in research published in the twentieth century. Antibody-mediated rejection (AMR), distinct from hyperacute rejection, emerged as a diagnostic concept in 1997 (2); subsequently it was recognized as a frequent cause of graft failure and an important cause of post-transplant complications (3–7). Affecting up to 25% of kidney allograft recipients (8, 9), the risk for AMR is low in the first year post transplantation in pre-transplant donor-specific antibody (DSA)-negative patients but reaches 30–40% in those who are DSA+. Beyond the first year following transplantation, risk for developing *de novo* (*dn*)DSA and subsequent AMR is associated with insufficient immunosuppression, which can result—among other factors—from non-adherence to standard-of-care regimens (10).

Advances in the development of sensitive assays for DSA identification have improved our understanding of AMR histopathology (11, 12). AMR is caused by antibodies that recognize donor human leukocyte antigen (HLA) on the kidney allograft endothelium, foreign to the recipient. Antibodies can also be formed against other allogeneic targets including non-HLA antibodies (e.g., against minor histocompatibility antigens) or non-allogeneic targets such as endothelial antigens or vimentin (13). DSA may develop before transplantation (because of blood transfusion, pregnancy, or previous allografts), or afterwards (*dn*DSA). AMR is recognized as a spectrum of discrete injury patterns, as outlined below.

## AMR in the Banff Classification

The detrimental impact of AMR on kidney transplantation outcome has been known for decades, as illustrated by the early routine implementation of crossmatching to avoid this rejection phenotype (14). The theoretical importance of AMR in kidney transplantation pathology was acknowledged at the first Banff meeting to focus on allograft pathology, in 1991 (15), However, this report only designated hyperacute rejection because of preformed DSA as a separate category (category 2) that was recognized as the most severe form of rejection, usually leading to immediate graft loss (15). In addition to hyperacute rejection, the 1997 update included delayed (accelerated acute) AMR and described histopathological and serological (crossmatch) diagnostic criteria (2). Reflecting the growing body of knowledge about AMR in kidney transplantation, diagnostic criteria and subcategories of AMR in Banff Classifications have changed considerably over time.

The next advancement followed the introduction of C4d staining, which documented histopathogenetic links between circulating DSA and organ damage, by detecting complement activation by DSA fixed to surface antigens on the endothelial cell (16). The 2001 Banff meeting recognized several histological types of acute/active (a)AMR, thereby expanding Category 2 diagnoses to include the following: 1) acute tubular necrosis-like minimal inflammation; 2) with capillary margination (glomerulitis and peritubular capillaritis [now considered microvascular inflammation, MVI]) and/or thromboses; and 3) with transmural arteritis and/or arterial wall necrosis. The reference to clinical presentations (“hyperacute” and “accelerated acute”) was abandoned, with emphasis shifting to histopathological features. Of note, all three AMR subtypes required C4d positivity (17).

Chronic (c)AMR subtypes were first recognized in the Banff 2005 update, as chronic active (ca)AMR, with transplant glomerulopathy (TG) and/or severe peritubular capillary basement membrane multilayering (PTCML) and/or simple interstitial fibrosis and tubular atrophy and/or arterial fibrous intimal thickening, with C4d positivity (18). Evidence of the pathogenetic link between aAMR and cAMR was discussed at the Banff 2017 meeting (19). The requirement for both DSA and C4d positivity to diagnose all subcategories of AMR (18) was relaxed in 2009, when subcategories for C4d−/DSA+ cases “suspicious for AMR” were created, matching the morphological patterns listed above but without C4d positivity (20). Further evidence (4) led to full recognition of C4d− AMR in the 2013 Banff update (21), and a diagnostic flowchart was created featuring subcategories “C4d positivity without evidence of rejection,” “suspicious for aAMR,” “aAMR,” “suspicious for caAMR,” “caAMR,” and “cAMR.” The flowchart accommodated numerous combinations of histopathological findings (21) that were simplified in the 2017 Banff update to form the categories listed in Table 1 (19, 22, 23). Most importantly, the “suspicious” categories were abandoned. Subsequently, only minor modifications have been made (22).

**TABLE 1:** Antibody-mediated changes (19, 22, 23); diagnostic criteria groups are used to reach one diagnosis.

Diagnosis	Diagnostic criteria groups
**C4d staining without evidence of rejection**	**Group 1: AMR activity**
• Lesion Score C4d > 1 (immunofluorescence on fresh frozen tissue) OR C4d > 0 (immunohistochemistry on paraffin-embedded tissue) AND	• Banff Lesion Score g > 0 in the absence of glomerulonephritis and/or Banff Lesion Score ptc>0 in the absence of TCMR or borderline changes
• Banff Lesion Scores t0, v0, no arterial intimal fibrosis with mononuclear cell inflammation in fibrosis and formation of neointima, no criterion from Group 1 (AMR Banff activity), no criterion from Group 4 (histologic features of AMR chronicity), no increased expression of thoroughly validated gene transcripts/classifiers in the biopsy tissue strongly associated with AMR	• Banff Lesion Score v > 0
	• Acute thrombotic microangiopathy in the absence of any other cause
	• Acute tubular injury in the absence of any other apparent cause
**Active AMR**	**Group 2: Antibody interaction with tissue Banff Lesion Score C4d > 1 (IF on fresh frozen tissue) or C4d > 0 (IHC on paraffin-embedded tissue)**
• No criterion from Group 4 (histologic features of AMR chronicity) AND	• At least moderate microvascular inflammation (g + ptc>1) in the absence of borderline changes (Diagnostic Category 3) or acute TCMR (aTCMR; Diagnostic Category 4). If borderline changes or aTCMR are present, Banff Lesion Score g + ptc>1 is not sufficient; g ≥ 1 is required
• ≥1 criterion from Group 1 (AMR activity) AND	• Increased expression of thoroughly validated gene transcripts/classifiers in the biopsy tissue strongly associated with AMR
• ≥1 criterion from Group 2 (antibody interaction with tissue) AND	
• ≥1 criterion from Criteria Group 3 (DSA or equivalent) AND	
**Chronic active AMR**	**Group 3: DSA or equivalent**
• ≥1 feature from Group 4 (histologic features of AMR chronicity) AND	• DSA (anti-HLA or other specificity)
• ≥1 criterion from Group 2 (antibody interaction with tissue) AND	• Banff Lesion Score C4d > 1 (IF on fresh frozen tissue) or C4d > 0 (IHC on paraffin-embedded tissue)
• ≥1 criterion from Group 3 (DSA or equivalent)	• Increased expression of thoroughly validated gene transcripts/classifiers in the biopsy tissue strongly associated with AMR
**Chronic AMR**	**Group 4: Histologic features of AMR chronicity**
• Banff 2017 permits the use of this term for biopsy specimens showing TG and/or PTCML in the absence of criterion of current/recent antibody interaction with the endothelium (Criteria Group 2) but with a prior documented diagnosis of active or chronic active AMR or documented prior evidence of DSA	• Banff Lesion Score cg > 0 (by light microscopy or EM, if available), excluding biopsies with evidence of chronic thrombotic microangiopathy
	• ≥7 layers in 1 cortical peritubular capillary and ≥5 in 2 additional capillaries, avoiding portions cut tangentially by EM, if available (severe PTCML); arterial intimal fibrosis of new onset, excluding other causes; leukocytes within the sclerotic intima favor chronic AMR if there is no prior history of biopsy-proven TCMR with arterial involvement, but are not required

AMR, antibody-mediated rejection; aTCMR, acute T cell-mediated rejection; DSA, donor-specific antibody; EM, electron microscopy; HLA, human leukocyte antigen; IF, immunofluorescence; IHC, immunohistochemistry; PTCML, peritubular capillary basement membrane multilayering; TCMR, T cell-mediated rejection; TG, transplant glomerulopathy.

Adapted from Roufosse et al., 2018 (23).

Currently, AMR diagnosis within Banff Classification category 2 is based on four partially overlapping components: histological features of AMR activity; evidence of antibody interaction with graft vascular endothelium; histological features of AMR chronicity; and DSA or equivalents (Table 1) (19, 21). Reaching an AMR diagnosis requires a combination of these criteria to be fulfilled.

## AMR and Allograft Outcome

Updates to the Banff Classification of AMR over time make it difficult to maintain long-term follow-up registries or compare literature. Moreover, the interobserver agreement (κ-statistic) of the most important lesion scores for AMR was quoted as 0.39 for Banff Lesion Score g, 0.38 for ptc, and 0.48 for cg—at best a fair-to-moderate agreement, even among very experienced transplant nephropathologists (24).

Several problems arise when reviewing evidence of an association between AMR and allograft outcome. Firstly, AMR definitions have changed very frequently since 2001, as outlined above, making it difficult to compare data from studies conducted over the last 2 decades. Secondly, Banff diagnostic criteria and categories are adjusted based on antecedent literature, and as they arise as a synthesis of several different studies, rarely fully align with literature on which they are based. Conversely, outcomes of AMR diagnoses according to their strict definitions in the Banff Classification have rarely been investigated for either their association with outcome, or their success in delineating which patients are eligible for a specific therapy; where this has been done, results show improved prediction of outcome with the 2013 version compared to the 2003/2007 Classification, and with the 2013 version compared to the 2017 Classification (25, 26). Instead, researchers often use slightly different definitions for the categories, with bespoke combinations of components and cut-offs for defining AMR, instead of the strict definitions last proposed by Banff. Thirdly, several Banff inclusion criteria for defining AMR are difficult to apply in clinical practice; this is one reason why precise Banff categories for AMR are rarely tested for their association with outcome. For example, cAMR categorization is mainly based on light microscopic features of Banff Lesion Score cg (TG), because detection of “severe PTCML” as an inclusion criterion for cAMR relies on electron microscopy (EM) (27). Few studies use arterial intimal thickening of new onset as an inclusion criterion for cAMR because it is difficult to score: this finding is dependent on arteries being cut transversally, is associated with unreliable arterial sampling (of both the current and previous biopsies), and in some cases is impossible to obtain because of lack of previous biopsies to use as a baseline. “Acute TMA” (thrombotic microangiopathy) is rarely the sole inclusion criterion for AMR, because it is hard to completely exclude TMA of other causes, it is rarely seen as an isolated feature without other features of AMR such as microcirculation inflammation, and because a Banff Working Group has yet to agree on a consensus definition of TMA (68).

To our knowledge, no method of transcriptome analysis has been formally recognized as thoroughly validated by Banff. No transplant centers have obtained adequate clinical validation to use transcript analysis for defining AMR, as required by Banff consensus. In addition, although the Banff Classification makes no distinction between AMR in patients with preformed DSA (high-risk) and non-sensitized (low-risk) transplant recipients, the diagnosis and treatment pathways for both groups might be quite different, as are the underlying biology and clinical phenotypes. Consequently, AMR classification may need to include more than histology, because identical histological diagnoses in the kidney (such as TG or TMA) can be the consequence of different disease entities (28). Finally, a diagnosis based on histology alone is not sufficient to describe the underlying pathophysiology. As suggested in the consensus report (28), for disease classification and outcome prediction, timing and clinical phenotype are crucial; and whether the patient has *dn*DSA, preformed DSA, or no HLA-DSA must also be known.

Thus, although the basic principles of diagnosing AMR have generally remained constant, given the considerable changes to Banff definitions of AMR, longitudinal comparison of literature findings is more challenging for AMR than for aTCMR. Interpretation of the AMR literature must be undertaken cautiously, taking account of these limitations. In reviewing evidence that could serve as background for the definition of AMR, first it is important to evaluate studies that have assessed outcomes associated with various combinations of biopsy features. In the following sections, we divide this information into subcategories broadly based on the Banff classification. After evaluating the literature on allograft outcome, we consider data relating to associations between outcome and individual biopsy features that are components of AMR (19). Moreover, it must be stated that we had to use the best available evidence for our consensus definitions of AMR. Inevitably, we had to omit rarer, insufficiently defined or researched phenotypes of the wide clinicopathological spectrum of AMR. Research should focus on diagnostic criteria for such rarer phenotypes, their outcome and their suitability for inclusion in AMR treatment studies. Of course, both the Banff Classification and future endpoint definitions will reflect any such evidence arising from these studies. In the interim, researchers are free to use their own endpoints. The choice of alternative endpoints is particularly justified in special scenarios such as in sensitized recipients requiring desensitization for transplantation.

## Banff Classification: AMR Subcategories as Endpoints

### C4d Staining Without Evidence of Rejection

This subcategory is discussed in conjunction with C4d positivity with acute tubular injury (ATI) in the absence of other apparent cause.

### Active AMR

Much of the evidence for an association between aAMR and outcome (i.e., graft loss) comes from publications that only include components of aAMR (e.g., MVI, C4d, and/or DSA) (Table 2) (29–34). Evidence of an association between aAMR and outcome derives from retrospective observational studies that rarely distinguish between pure aAMR and caAMR; thus, few studies specifically investigate the relationship between a Banff subcategory aAMR diagnosis and outcome.

**TABLE 2:** Studies investigating associations between Banff diagnostic category “active AMR” and outcome (29–34).

References	Endpoint	Definition of aAMR	Study type	Cohort	Findings	Level of evidence (grade)
Solar-Cafaggi et al. (29)	Graft loss	Mixed (unseparated) aAMR and caAMR (Banff 2007 or 2017 criteria); indication and protocol biopsies; incomplete DSA data	Single-center retrospective	N = 201	Increased graft loss (*p* = 0.001)	Very low
Sai et al. (30)	sCr × 1.5; cAMR and graft loss	Banff 2013	Retrospective	N = 627 protocol + indication biopsies; C4d+ AMR (*n* = 24) and C4d− AMR (*n* = 20) vs. controls (*n* = 20) AMR−	Significantly more cAMR and graft loss on follow-up (between-group analysis [Mann−Whitney], not outcome analysis)	Very low
Orandi et al. (31)	Graft loss	Banff 2013 Methods do not clarify whether cases with cg are included	Retrospective	N = 217 patients with AMR; (142 clinical AMR, 77 subclinical) + controls (426 clinical, 231 subclinical); high proportion (63%) of HLA-incompatible transplants, so may not apply to conventional transplantation	Graft loss in subclinical AMR 2.15-fold (95% CI 1.19–3.91; *p* = 0.012) higher than for matched controls without AMR; graft loss clinical AMR 5.79-fold (95% CI 3.62–9.24; *p* < 0.001) higher than for matched controls without AMR	Low +1 (RR > 5 for clinical AMR)
Orandi et al. (32)	TG and graft loss	Banff 2013 aAMR and/or caAMR likely both included	Single-center, retrospective; all biopsies in 1st year post transplant (indication + protocol)	51 C4d− and 156 C4d+ cases of AMR	TG risk same in C4d− and C4d+ but not vs. controls; 1-year and 2-years post-AMR graft survival: C4d− vs. controls: 2.56-fold (95% CI 1.08–6.05; *p* = 0.033); C4d+ vs. controls 3.70-fold (95% CI 2.47–5.54; *p* < 0.001); no difference between C4d− and C4d+	Low
Everly et al. (33)	Graft loss	Altered Banff definition used: Banff 2003 AMR including suspicious (i.e., if ≥ 2 of the following present: DSA, histopathologic changes consistent with AMR and C4d+ staining in PTC ± other structures); do not specify active or chronic active	Retrospective	Patients with acute cellular rejection (*n* = 30) or AMR (*n* = 30)	Significantly worse survival in AMR (*p* < 0.001)	Low
Loupy et al. (34)	TG; 1/sCr and eGFR	Altered Banff definition used (Banff 1997 + addition of C4d− AMR); contains some cg/chronic cases at baseline	Retrospective	Pre-sensitized patients (*n* = 54)	Subclinical AMR at 3 months associated with interstitial fibrosis and tubular atrophy, TG and worse function at 1 year	Low

a, acute/active; AMR, antibody-mediated rejection; c, chronic; ca, chronic active; CI, confidence interval; DSA, donor-specific antibody; eGFR, estimated glomerular filtration rate; HLA, human leukocyte antigen; PTC, peritubular capillaries; sCr, serum creatinine; TG, transplant glomerulopathy.

Given the heterogeneity of definitions, overall, the quality of evidence that strictly defines the association between aAMR and increased graft loss is low; in recipients of HLA-incompatible grafts, quality of evidence is higher. However, if one evaluates the literature with less stringency about the exact AMR definition, there is consensus that aAMR is an important risk factor for graft failure (10, 28). Moreover, in the era of powerful T cell inhibition as standard immunosuppression, outcome after aAMR at time of graft dysfunction is significantly worse than outcome after aTCMR (35).

In the absence of dysfunction (i.e., subclinical AMR in protocol biopsies), the outcome used for features of aAMR is usually TG rather than graft loss, although a retrospective study indicated that subclinical AMR in 1-year protocol biopsies had a detrimental impact on graft survival (36). There is general agreement on treating aAMR regardless of whether graft dysfunction occurs, further illustrating the clinical relevance of this phenotype (28). This is discussed further in the section below, “Subclinical AMR Including Incomplete Phenotypes.”

Our proposal is that aAMR, exactly as defined by the current Banff classification, cannot be adopted as a surrogate endpoint for future cAMR and graft loss in low-risk situations, i.e., non-sensitized graft recipients, without DSA against the graft. Conversely, in high-risk patients, i.e., sensitized patients with DSA against the graft, evidence supports features of aAMR as a surrogate for graft loss, especially if associated with graft dysfunction. Future research should aim to establish outcome (graft loss, graft function, future cAMR, or caAMR) in patients with aAMR, strictly defined according to Banff criteria and specifically excluding cases with features of chronicity. Such research should involve retrospective and prospective studies, and high- and conventional-risk transplantations. Data from randomized controlled trials investigating aAMR treatment regimens would also be particularly valuable. Although further data are awaited, there is broad consensus on the clinical relevance and impact of aAMR after kidney transplantation. Since aAMR leads to therapeutic interventions, treatment burden, associated morbidity, and increased cost, features of aAMR represent a key endpoint for interventional studies.

### Chronic AMR and Chronic Active AMR

According to the 2017 Banff Classification, a diagnosis of cAMR or caAMR can only be established based on presence of TG (Banff Lesion Score cg > 0) and/or severe PTCML. For caAMR, this must be accompanied by “evidence of antibody interaction with tissue” and “DSA or equivalent”; for cAMR, this must be in conjunction with “a prior documented diagnosis of aAMR or caAMR or documented prior evidence of DSA.” The ill-defined transplant vasculopathy is no longer considered a chronicity parameter for these diagnoses (19). Data for patients fulfilling identical Banff criteria of severe PTCML as the indicator of AMR chronicity, in conjunction with solid-phase DSA testing, are scant. Therefore, we present evidence only for outcomes in patients with TG, with “histological lesions strongly associated with AMR” (i.e., MVI ≥2, C4d positivity, or “increased expression of thoroughly validated gene transcripts/classifiers in biopsy tissue”) (Table 3) (37–54). In reviewing literature on cg, as with the other histological lesions, caution should be taken because of the relatively limited interobserver agreement (24).

**TABLE 3:** Studies investigating associations between Banff diagnostic categories cAMR/caAMR and outcome; and/or investigating TG (37–54).

References	Endpoint	Parameter investigated	Findings	Level of evidence (grade)
Lesage et al. (37)	Graft loss	GBM splitting	∼50% graft loss within 24 months after index biopsy; HR even after adjustment for sCr and proteinuria >5 vs. controls	Moderate
Wavamunno et al. (38)	Death-censored transplant survival	Ultrastructural	No difference in death-censored transplant survival between 7 patients with Banff cg ≥ 1 (Banff 1997) within the first 5 years post transplantation vs. 8 controls	Very low
Perkowska-Ptasinska et al. (39)	Transplant survival in subgroups of TG	Banff cg ≥ 1 Banff 2007 = Banff 1997	38/158 patients with TG lost transplant within 98 months (range, 3–215 months); no control cohort; extraction of outcome data on c/caAMR/TG not possible	Not applicable
Shimizu et al. (40)	Graft loss in patients with a diagnosis of TG	Banff cg ≥ 1 Banff 2009 = Banff 1997	22% graft loss within observation time (time unclear); includes ABO-incompatible transplants; Unclear whether Banff chronic AMR fulfilled; no comparison with controls regarding outcome	Not applicable
Hayde et al. (41)	Death-censored transplant survival	cAMR compared to IFTA and TG (authors’ own definitions; invalid criteria for TG: “by electron microscopy … electron-lucent widening of the subendothelial zone of the GBM, subendothelial accumulation of flocculent material, with or without a new subendothelial basement membrane layer”)	cAMR associated with significantly lower graft survival compared with IFTA (*p* = 0.01) but not compared with TG	Low
Pefaur et al. (42)	Transplant survival	Unclear criteria for TG	Retrospective study, 3 patients; extraction of outcome data on c/caAMR/TG not possible	Not applicable
Shimizu et al. (43)	Transplant survival	Banff cg ≥ 1 Banff 2007 = Banff 1997	Retrospective study, 13 patients, no control group; 2/13 grafts lost; unclear observation time; extraction of outcome data on c/caAMR/TG not possible	Not applicable
John et al. (44)	Death-censored transplant survival	Banff cg ≥ 1 Banff 1997	Retrospective study, 36 patients with TG, 5-years death-censored graft loss 16.7%; no control group; extraction of outcome data on c/caAMR/TG not possible	Not applicable
Nair et al. (45)	Graft loss	Unclear criteria for TG that do not necessarily involve GBM splitting	Three patients with TG within first 6 months post transplantation; no control group; extraction of outcome data on c/caAMR/TG not possible	Not applicable
Lopez Jimenez et al. (46)	Graft loss	Banff cg ≥ 1 Banff 1997	Retrospective study, 30 patients with TG; 50% graft loss mean 25 ± 20 months post biopsy; no control group; extraction of outcome data on c/caAMR/TG not possible	Not applicable
Kamal et al. (47)	Graft loss	Banff cg ≥ 1 Banff 2007 + 2009 = Banff 1997	Retrospective study, 52 patients with TG, 17 (32%) with graft loss, median time to graft loss 16 months, no control cohort; extraction of outcome data on c/caAMR/TG not possible	Not applicable
Dobi et al. (48)	Transplant survival	Banff cg ≥ 1a Banff 2013	Retrospective analysis, 57 patients with TG; no control cohort; extraction of outcome data on c/caAMR/TG not possible	Not applicable
Halloran et al. (49)	Death-censored transplant survival	Banff cg ≥ 1a Banff 2013	Retrospective analysis, 27 patients with TG; extraction of outcome data on c/caAMR/TG not possible	Not applicable
Toki et al. (50)	No outcome data (eGFR at time of biopsy)	Banff cg ≥ 1b Banff 2013	Retrospective analysis, 127 patients with TG; extraction of outcome data on c/caAMR/TG not possible; no outcomes data	Not applicable
Courant et al. (51)	Death-censored transplant survival	cAMR according to Banff 2013 with DSA+	Retrospective data, 9 patients with cAMR; extraction of outcome data on c/caAMR/TG not possible	Not applicable
Lubetzky et al. (52)	Transplant survival	Banff cg ≥ 1 Banff 1997; patients with TG and DSA+/MVI−	Retrospective analysis, 24 patients 50% graft loss in 3 years; extraction of outcome data on c/caAMR/TG not possible	Not applicable
Sablik et al. (53)	Transplant survival	Banff cg ≥ 1b Banff 2015	Retrospective analysis, 41 patients with caAMR; no control cohort; extraction of outcome data on c/caAMR/TG not possible	Not applicable
Aubert et al. (54)	Transplant survival	Banff 2009, 2011, 2013. Unclear if cg1a included	Retrospective analysis, 385 patients with TG; different immunological, functional and histological TG subtypes described; no comparison to control group without TG; extraction of outcome data on c/caAMR/TG not possible	Not applicable

a, acute/active; AMR, antibody-mediated rejection; c, chronic; ca, chronic active; DSA, donor-specific antibody; eGFR, estimated glomerular filtration rate; GBM, glomerular basement membrane; HR, hazard ratio; IFTA, interstitial fibrosis and tubular atrophy; MVI, microvascular inflammation; sCr, serum creatinine; TG, transplant glomerulopathy.

A retrospective study of 44 patients with TG examined the outcome of graft loss, with TG defined as Banff cg > 0 (glomerular basement membrane splitting of >10% of the entire tuft in the most severely affected glomerulus) (2); this definition remained relevant until Banff 2011 (55). With this TG threshold—higher than the current threshold of glomerular basement membrane “double contours (incomplete or circumferential) in at least three glomerular capillaries by EM, with associated endothelial swelling and/or subendothelial electron-lucent widening” (19, 23)—the publication reported ∼50% graft loss within 24 months after the index biopsy. There appears to be no difference in outcome between cases with C4d positivity and DSA negativity (qualifying as caAMR according to Banff 2017/2019) and TG cases with C4d negativity and DSA positivity (37) (qualifying as cAMR or caAMR if moderate MVI is present, according to Banff 2017/2019) (19, 20, 23).

To investigate associations between TG and other parameters, as well as with outcomes, using archetypal analysis, a retrospective study of 385 patients with TG identified five distinct immunological, histological, and functional profiles of TG that were associated with allograft failure (54). Another retrospective analysis of TG in 954 kidney transplant recipients (3744 biopsies) found that TG occurred in >75% of the patients in the absence of HLA-DSA, independent of HLA molecular mismatches; it represented a different phenotype that had lower levels of concomitant inflammation and graft loss compared with HLA-DSA+ TG (56). An additional recent retrospective study found that proteinuria, C4d presence, and mesangial matrix expansion were important for outcome, while other histological markers (e.g., Banff Lesion Score cg) were not (57).

Because of repeated revisions to Banff criteria (including gene transcripts and the requirement for EM, to detect PTCML and early TG lesions), the incidence of caAMR is under-reported. No studies fulfill all criteria for this diagnosis according to Banff 2017 or have a sufficient follow-up to use strictly defined Banff caAMR as an endpoint (58).

We therefore recommend that clinical trials in kidney transplantation using caAMR as an endpoint or an inclusion criterion strictly adhere to Banff consensus criteria and report granular histological features of Banff Lesion Scores, to allow between-trial comparisons. Additional research is needed, in high- and conventional-risk transplantation scenarios that consider the effect of treating aAMR earlier, equally defined according to the strict Banff Classification.

### Suspicious for AMR Subcategories

A noteworthy change to the Banff Classification in 2017 was its omission of ‘suspicious for aAMR’ and ‘suspicious for caAMR’ categories (19). The most frequent reason leading to a diagnosis of ‘suspicious for “aAMR” instead of “aAMR” was absence of evidence for DSA or C4d positivity (9).

Until 2019, no publication presented outcomes for patients with “suspicious for aAMR.” Then, the evidence appeared, with a caveat, because the 123 DSA− patients with AMR included six patients with TG (Banff Lesion Score cg ≥ 1); irrespective of C4d status, outcomes for patients with histological features of AMR but without DSA were no different than for controls without AMR (59). Although there was a significant association between C4d status and DSA in this study, C4d and DSA were not interchangeable (accuracy of C4d deposition for DSA positivity was 59–65%) (59).

The literature offers even less information about the diagnostic subcategory of “suspicious for caAMR,” eliminated from Banff in 2017. One study involving 21 DSA− patients showed an average transplant survival after diagnosis of 3.7 years (53).

Some DSA− cases “suspicious for AMR” could be explained by non-HLA antibodies. Without any hard evidence, standardized tests, or validated assay and cut-off value to screen for non-HLA-DSA, we do not recommend that non-HLA-DSA be considered in the diagnosis of AMR. Further research is needed before non-HLA antibodies can be included in the definition of endpoints for registration studies.

Overall, we do not recommend using cases in the “suspicious for” categories as endpoints.

## Individual Histopathological Features of AMR as Endpoints

### “ATI in the Absence of Any Other Apparent Cause” as a Feature of AMR, in Conjunction with C4d Positivity and DSA

This section reports on two category 2 diagnoses that are separated in the Banff 2017/2019 Classification (19,21). Firstly, aAMR, where evidence of tissue injury is only “ATI in the absence of any other apparent cause” (ATI-AMR); to diagnose aAMR in such cases, C4d must be positive. Secondly, “C4d-staining without evidence of rejection”: this is a subcategory of “antibody-mediated changes.” Evidence relating to both entities is reviewed together, because the difference between them relates to presence or absence within the biopsy of the Banff additional diagnostic parameter “ATI in the absence of any other apparent cause.” ATI has not been redefined since the 1995 Banff meeting; most transplant biopsies show a mild degree of ATI that might not qualify for ATI-AMR; at the lower end of the spectrum of ATI severity, the difference between ATI-AMR and C4d+ without evidence of rejection is tenuous. We are not aware of an evidence-based definition separating ATI-AMR and ATI of other causes.

Technically, according to the Banff classification, a biopsy that is C4d+ with DSA but with a reasonable other cause of ATI (e.g., ischemia/reperfusion injury) is not AMR, yet publications have not assessed for (or reported on) other causes of ATI. Some early reports on C4d staining date from before widespread recognition of the full spectrum of histological features of aAMR, therefore descriptions of poor outcomes for C4d+ cases “without features of rejection” must be handled cautiously.

The 2001 Banff meeting recognized a form of AMR with no or little inflammation, included in the list of category 2 diagnoses as “acute tubular necrosis-like minimal inflammation, C4d+.” It was stated that “acute humoral rejection may be manifested only by ATI without other evidence of rejection (seen in 10% of cases).” The evidence cited (60) describes two cases of AMR where ATI-like changes were the sole feature. It is likely that inclusion of the ATI-AMR in Banff 2001 was based on the combined experience of meeting attendees, from an era when less sensitive pre-transplant evaluation for HLA antibodies created a population of accelerated/acute AMR with these features. Current data on the incidence of this histological variant, in both low- and high-risk transplantations, are lacking.

The 2007 Banff meeting (18) described a different subcategory of antibody-mediated changes that is now called “C4d staining without evidence of rejection” (19). It includes cases with C4d+ staining, but no features of activity or chronicity related to AMR (Table 1), and no features of TCMR or borderline changes. Banff 2017 further specified that there should be no evidence of increased expression of thoroughly validated gene transcripts or classifiers in biopsy tissue samples strongly associated with AMR (19). This diagnosis excludes cases with “ATI in the absence of any other apparent cause,” although—as stated above—it is likely that mild ATI features are frequently observed. This category includes biopsies from recipients of ABO-incompatible transplants, in which it is associated with good outcomes (19), but also includes cases from recipients of ABO-compatible transplants, in which case its significance is unclear.

Additional publications investigating the link between ATI-AMR and C4d+ without evidence of rejection, with outcome data, are presented in Table 4 (61–66). These studies provide low-quality evidence, but further research might have an impact on confidence in the estimate and could change the assessment. The data suggest that, in sensitized patients, C4d+ ATI (likely severe) in the early post-transplant phase could represent early AMR and be associated with graft loss (61–63), whereas the significance of C4d+ with mild ATI in later post-transplant biopsies is less clear. Some evidence suggests it is not strongly associated with future AMR or graft loss (64–66).

**TABLE 4:** Studies investigating ATI-AMR and C4d+ without evidence of rejection (61–66).

References	Endpoint	Definition of Banff phenotype	Findings	Level of evidence (grade)
Haas et al. (61)	AMR	C4d+ in early post-reperfusion biopsies	Predicts future AMR (*n* = 2 positive crossmatch patients with later AMR)	Low
Djamali et al. (62)	AMR	C4d+ in early post-reperfusion biopsies; mild to moderately sensitized transplant recipients	Predicts future AMR	Low
Kikic et al. (63)	Graft loss	Biopsies with C4d; 42% of patients in the C4d+ group were pre-sensitized; mean time to biopsy in C4d+ group 0.75 mo	C4d associated with graft loss independently of presence of histological features of AMR; HR 1.85 (*p* < 0.0001)	Low
Nickeleit et al. (64)	Benefit from antirejection therapy	C4d+ with mild allograft dysfunction and no histological evidence of rejection	C4d+ with mild allograft dysfunction and no histological evidence of rejection does not benefit from antirejection therapy	Low
Dickenmann et al. (65)	Improved function after treatment	C4d+ biopsies without other histopathological features of AMR	Function improves in this group after treatment	Low
Dominy et al. (66)	AMR	C4d+ without evidence of rejection; mild ATI at most	Rather than histological features or DSA, transcript analysis for AMR signature distinguishes minority at risk of subsequent AMR	Low

AMR, antibody-mediated rejection; ATI, acute tubular injury; DSA, donor-specific antibody; HR, hazard ratio.

It is impossible to give a guideline recommendation because of inconsistent findings in the argument that C4d+ ATI without evidence of rejection is associated with increased risk of graft loss. In addition, there is no recent consensus definition of ATI, or of degrees of severity of ATI, or of what reasonably constitutes exclusion of other causes of ATI. Therefore, we recommend that the C4d+ ATI-only form of AMR and C4d+ without evidence of rejection subcategory of AMR should not be used as an efficacy measure in clinical trials. We also recommend that future research incorporates definitions of ATI and assessments of its severity, based on definitions agreed in the context of international collaborations (e.g., Banff Working Group for Rules and Dissemination). Such research should include both patients with preformed antibodies (sensitized) and non-sensitized patients, with representation of early and late post-transplant periods.

### Endarteritis

Endarteritis is also a feature of aTCMR that initially was not included in AMR definitions; this makes findings from early studies difficult to interpret for the given purpose (Banff Lesion Score v in “acute TCMR,” “mixed acute TCMR-AMR,” “pure AMR”). Although endarteritis was described as a risk factor for graft loss (67), there are insufficient published data on endarteritis in pure aAMR as an isolated histopathological finding to recommend its use as an AMR-related endpoint.

### Acute TMA in the Absence of Any Other Cause

Banff acknowledges that TMA can have a variety of causes in kidney transplant recipients (e.g., recurrent disease, infection, antiphospholipid antibodies, medication toxicity). During the 2015 Banff meeting, a working group was formed (68) to help with histopathological characterization of TMA in kidney transplantation. This group aimed to guide the development of precise diagnostic algorithms, including the creation of rules on how other apparent causes could be excluded, allowing for a *bona fide* diagnosis of AMR-associated TMA. In some patients with *dn*TMA, an underlying genetic defect in complement regulation might be relevant, although only one case series suggested this (69).

We are unaware of sufficient published data about the outcomes of adequately investigated cases of AMR-associated TMA. The largest case series describes 33 patients with TMA and C4d positivity, 40% of whom experienced transplant failure within 2 years of diagnosis (70). Since C4d positivity in peritubular capillaries and medullary vasa recta is extremely rare in native kidneys with TMA (71), this combination of findings can be considered “AMR-associated TMA,” as is currently the case according to Banff 2017 (19). However, the problem persists of excluding other causes of TMA. Nevertheless, for reasons outlined above, we would not encourage the use of TMA as isolated histopathological finding as an efficacy measure for clinical trials, in a context that does not meet the Banff diagnostic criteria for a full AMR phenotype. Nor is there enough evidence to recognize “acute TMA in the absence of any other cause” as a sufficiently robust criterion for aAMR.

### Microvascular Inflammation

MVI is the main histological feature indicating activity in aAMR and caAMR. The Banff criteria for AMR use cut-off values of MVI >0 and >1, respectively, to establish C4d+ and C4d− aAMR; these values were established by consensus, based on published evidence (19). MVI above a certain threshold in diagnostic biopsies is an independent predictor of graft loss and chronic lesions (Table 5) (72–77), although the quality of evidence is low. Moreover, low interobserver agreement in the exact grading of the underlying g and ptc lesions (24) suggests caution when using this parameter as an endpoint in studies.

**TABLE 5:** Studies investigating the association between MVI and outcomes (72–77).

References	Endpoint	Predictor	Findings	Level of evidence (grade)
Haas and Mirocha (72)	TG	MVI + endothelial lesions on EM	Indication biopsies (DSA at time of biopsy): MVI + endothelial lesions on EM associated with TG	Low
Bagnasco et al. (73)	TG	g	Patients with pre-transplant DSA (deceased donors including ABO-incompatible donors): g in protocol + indication biopsies associated with TG (*p* < 0.0001)	Low
Einecke et al. (74)	Graft loss	MVI	In multivariate analysis (indication + protocol biopsies, DSA post-transplant, living + deceased donors), graft failure correlated with MVI and scarring, but C4d staining was not significant	Low
Sis et al. (75)	Graft loss	MVI	Indication biopsies (anti-HLA antibodies at time of biopsy, living + deceased donors): g + ptc predicted graft failure independently of time, C4d and transplant glomerulopathy (*p* < 0.001)	Low
Verghese et al. (76)	Graft loss	MVI	Retrospective, no data on DSA, includes mixed TCMR-AMR indication biopsies. In indication biopsies carried out <1 year post-transplant, MVI associated with decreased death-censored graft survival, independent of the presence of C4d (*p* = 0.005)	Low
de Kort et al. (77)	Graft loss	MVI	Retrospective cohort study indication biopsies of patients with *dn*DSA: severe MVI >21-fold increased risk of graft failure (95% CI 2.5–180.0; *p* = 0.005), while C4d positivity on indication biopsy lost significance	Low

AMR, antibody-mediated rejection; CI, confidence interval; *dn*, *de novo*; DSA, donor-specific antibody; EM, electron microscopy; HLA, human leukocyte antigen; MVI, microvascular inflammation; TCMR, T cell-mediated rejection; TG, transplant glomerulopathy.

Based on the low-quality evidence that MVI is an independent predictor of graft loss, we cautiously recommend that the MVI score is used as an efficacy marker for clinical trials in kidney transplantation. We also recommend that further research is undertaken to confirm the effect of MVI on outcome, in prospective randomized controlled trials, with granular histological data for Banff Lesion Scores and DSA.

### C4d Positivity

There are caveats to the prognostic value of C4d status: thresholds for positivity scoring differ, depending on antibody and study. For example, the monoclonal antibody used on frozen tissue is particularly sensitive; therefore >10% of PTC must be positive, whereas for the polyclonal anti-C4d antibody used on formalin-fixed paraffin-embedded tissue samples, any percentage and intensity of PTC positivity sufficiently describes a biopsy as C4d+ (23).

Literature findings related to the potential prognostic value of C4d status are varied, likely because of the dynamic process (Table 6) (3, 63, 78, 79). A large body of evidence indicates that MVI is a better prognostic factor than C4d (74–77). C4d positivity as an isolated histopathological finding therefore cannot be recommended as an efficacy measure for clinical trials in kidney transplantation.

**TABLE 6:** Transplantation studies that feature C4d and outcomes (3, 63, 78, 79).

References	Endpoint	Predictor	Findings	Level of evidence (grade)
Naesens et al. (3)	Graft loss	Composite	Retrospective (indication biopsies, no info on DSA): C4d, TG, ongoing interstitial inflammation, dn/recurrent glomerular disease, IFTA significantly and independently associated with post-biopsy graft survival (MVI highly significant in univariate, but not retained in final multivariate model)	Low
Kikic et al. (63)	Graft loss	C4d	C4d associated with graft loss independently of the presence of histological features of AMR	Low
Sapir-Pichhadze et al. (78)	Graft loss	C4d	Systematic review (3492 abstracts: 3485 indication and 868 protocol biopsies). C4d+ associated with inferior allograft survival compared with DSA or histopathology alone	Low
Matas et al. (79)	Graft loss	C4d	Cross-sectional (retrospective) cohort (indication biopsies, DSA at time of biopsy, living + deceased donors): C4d−/DSA- recipients had significantly better (and C4d+/DSA+ worse) death-censored graft survival than other groups. C4d+/DSA− and C4d−/DSA+ had similar intermediate death-censored graft survival	Low

AMR, antibody-mediated rejection; *dn*, *de novo*; DSA, donor-specific antibody; IFTA, interstitial fibrosis and tubular atrophy; MVI, microvascular inflammation; TG, transplant glomerulopathy.

### Transplant Glomerulopathy

As TG is the main feature indicating chronicity in the diagnosis of caAMR and cAMR, much of the evidence indicating that this feature is an indicator of outcome has been covered above (Table 3). As with all individual histological lesions, moderate interobserver agreement in the graded scoring (24) suggests trial results should be interpreted cautiously.

In 55 patients with TG (Banff Lesion Score cg ≥ 1b) (19) there was a high risk of death-censored transplant survival in a multivariate analysis (HR 6.2; 95% CI 2.5–14.7; *p* < 0.0001) (80). Similar results were obtained in another multivariate analysis of 77 indication biopsies (HR 2.40; 95% CI 1.25–4.60; *p* < 0.01); this study used a lower threshold (Banff Lesion Score cg > 0) (21), equivalent to Banff 2017 (19, 23), but did not mention how many biopsies were examined with EM (51).

Applying Racusen’s criterion for defining glomerular basement membrane splitting, mentioned above (2), which has a higher threshold for TG than current criteria (19,23), Torres *et al.* identified ∼50% graft loss within 3 years after the index biopsy (81). Using the same threshold for TG, a retrospective study found graft loss in 2/12 patients with isolated TG in the absence of sufficient MVI, C4d positivity, or DSA positivity at the time of biopsy; notably, their definition did not necessarily exclude caAMR according to Banff 2017 (23, 68). While glomerular basement membrane splitting is a prerequisite for diagnosing TG as a manifestation of cAMR, it is by no means specific to AMR and can arise in different conditions—some of which are recognized by Banff—including TMA of causes other than AMR, *dn* or recurrent glomerulonephritis (23), hepatitis C virus infection (82), or hypertensive glomerulopathy (83). We recommend that further research is performed to establish the causes and impact of isolated TG that does not fulfill criteria for cAMR or caAMR. TG as an isolated histopathological finding cannot be recommended as an efficacy measure for clinical trials in kidney transplantation.

### Peritubular Capillary Basement Membrane Multilayering

Normal peritubular capillaries have a single basement membrane under the endothelial cell, and PTCML is characterized by an increase in basement membrane layers. Low levels of PTCML are seen in several conditions, whereas severe PTCML is a defining feature of chronicity in the Banff definition of AMR. Severe PTCML is characterized as seven or more layers of basement membrane in at least a single cortical peritubular capillary, and five or more layers in at least two additional capillaries.

Currently, PTCML is only diagnosed in transplantation biopsies by EM evaluation, which limits its use to centers sufficiently resourced to undertake such examinations. Even within EM-capable centers, this diagnostic method may be reserved for cases for which there is an indication [defined in Banff 2013 (21)]. There is therefore an inherent bias in reports investigating PTCML, which generally do not involve systematic assessment of all biopsies.

The limited number of observational studies investigating the link between PTCML and outcome (Table 7) (48, 84–86) provide low-quality evidence: further research is likely to have an impact on confidence in the estimate and may indeed change it. Although there is consistent evidence that PTCML is associated with future TG and increased risk of graft loss, it is impossible to give a guideline recommendation or consensus-based statement, because studies use different methodologies. Therefore, we recommend that PTCML as an isolated histopathological finding is not used as efficacy measure for clinical trials. We also recommend that future research incorporates methods of counting basement membrane layers that are agreed in the context of international collaborations (e.g., Banff Working Group for Electron Microscopy), assess non-selected populations of transplant biopsies, and utilize clinically meaningful scoring systems that predict graft loss and cAMR development.

**TABLE 7:** Studies that feature PTCML assessment and outcomes (48, 84–86).

References	Endpoint	Definition of PTCML	Findings	Level of evidence (grade)
Einecke et al. (84)	Graft loss	One PTC with ≥5 basement membrane layers	In non-selected transplant population, 1 PTC with ≥5 basement membrane layers predictive of graft loss in multivariate analysis (HR 1.98, *p* = 0.01)	Low
Roufosse et al. (85)	TG	Numbers of PTC with ≥3 and ≥5 basement membrane layers	Risk of TG increases with increasing numbers of PTC with ≥3 and ≥5 basement membrane layers	Low + 1 (‘dose–response’ gradient)
de Kort et al. (86)	Graft loss	Three PTC with ≥5 basement membrane layers	In patients with *dn*DSA, 3 PTC with ≥5 basement membrane layers associated with increased graft loss (*p* = 0.016)	Low
de Kort et al. (86)	TG	Mean basement membrane layer count >2.5	Mean PTCML count >2.5 associated with increased risk of TG (*p* = 0.001); progressors to >2.5 associated with more TG	Low
Dobi et al. (48)	Graft loss	PTC circ score ≥3	In patients with cAMR, PTC circ ≥3 predicts graft loss	Low

AMR, antibody-mediated rejection; c, chronic; *dn, de novo*; DSA, donor-specific antibody; HLA, human leukocyte antigen; HR, hazard ratio; PTC, peritubular capillary; PTCML, peritubular capillary basement membrane multilayering; TG, transplant glomerulopathy.

### Transplant Vasculopathy

The definition of transplant vasculopathy as evidence of AMR chronicity remains ambiguous in the Banff Classification (19, 23). Consequently, using the sole finding of transplant vasculopathy cannot be encouraged as an efficacy measure for clinical trials. We are unaware of any studies reporting outcomes for patients with this criterion for AMR chronicity. Older publications discussing the impact of Banff Lesion Score cv without specification of morphological details of this finding (88) are unhelpful, because this score can be influenced by factors other than AMR and can be ≥ 1 even in implantation biopsies (donor-derived).

### Increased Transcripts or Transcript Sets Strongly Associated with AMR

Increased expression of thoroughly validated gene transcripts/classifiers in biopsy tissue strongly associated with AMR provides evidence of current/recent antibody−tissue interactions, according to the Banff 2017/2019 definition of AMR (19, 22). Notably, many publications relating to transcript analysis do not distinguish between TCMR and AMR, which limits the studies that can be included here. Retrospective investigations of the link between gene transcripts/classifiers strongly associated with AMR and outcome also provide low-quality evidence (Table 8) (4, 47, 88–91). However, the INTERCOM study prospectively analyzed 300 transplantation biopsies (264 patients) and found that assigning an AMR score based on molecular analysis identified signs of AMR in 41% of biopsies where AMR had not been suspected: the score also showed a better correlation with graft failure than conventional assessments (92). The MMDx Kidney study group also prospectively collected microarray data from >1200 transplant biopsy samples and found that precision microassessment enabled six archetypes to be generated (from no rejection through TCMR and all stages of AMR) (93). Further research could have an impact on confidence in the estimate and might change it.

**TABLE 8:** Studies that feature ‘Evidence of gene transcripts/classifiers strongly associated with AMR’ and outcomes (4,47,88–91).

References	Endpoint	Definition of molecular marker	Findings	Level of evidence (grade)
Sellares et al. (4)	Graft loss	AMR classifier	Retrospective cohort, 315 patients; AMR classifier predicts graft loss in Cox multivariate analysis	Low
(87)	Graft loss and progression of chronic injury	AMR molecular score and endothelial DSA-selective transcripts	2 cohorts (principal *n* = 74, validation *n* = 54) with cases of AMR in 1st year after transplant (early AMR); AMR Molecular Score (HR 2.22; 95% CI 1.37–3.58; *p* = 0.001) and endothelial donor-specific antibody-selective transcripts (HR 3.02; 95% CI 1.00–9.16; *p* < 0.05) independently associated with increased risk of graft loss	Low
Loupy et al. (88)				
Yazdani et al. (89)	Graft loss	Differential expression of 503 unique genes in AMR, with significant enrichment of NK cell pathways	Retrospective cohort, with validation in external cohort for outcome analysis; microarray transcriptomic data from case–control study (*n* = 95) to identify genes differentially expressed in AMR; multivariate Cox analysis: NK cell gene signature predicted graft loss better than (*p* < 0.001), and independent of, the diagnosis of rejection according to Banff (*p* = 0.039)	Low
Sis et al. (90)	Graft loss	ENDATs	Retrospective cohort of indication biopsies validated in independent set; microarray analysis for ENDATs. Many individual ENDATs were increased in AMR and predicted graft loss; high ENDAT score in patients with DSA predicts graft loss (but no increase in graft loss if DSA− and ENDAT+)	Low
Dominy et al. (91)	Graft loss	*Sh2D1b* and *Mybl* score	Retrospective cohort of 57 biopsies from patients with AMR or normal surveillance biopsies; 2-gene signature predicts graft loss in whole group and within DSA+ group	Low
Kamal et al. (47)	Graft loss; no formal outcome analysis	Various gene expression levels	Retrospective cohort of patients with TG; significantly increased levels endothelial cell–associated transcripts, gene transcripts associated with complement cascade, interleukins and their receptors, and granulysin in patients with graft loss	Very low

AMR, antibody-mediated rejection; CI, confidence interval; DSA, donor-specific antibody; ENDAT, endothelial cell–associated transcript; HR, hazard ratio; NK, natural killer; TG, transplant glomerulopathy.

Although there is consistent evidence that gene transcripts/classifiers strongly associated with AMR are associated with graft loss (and in some cases, the evidence comes from multivariate analyses with validation groups), it is impossible to give a guideline recommendation or consensus-based statement. Different gene sets/classifiers are used across the studies, with no unifying set of genes agreed on for future validation in prospective research. Also, to our knowledge, no transplant centers have clinical validation for use of transcript analysis for AMR, especially for improving the prediction of graft outcome. Consequently, we recommend that gene transcripts/classifiers strongly associated with AMR are not used as efficacy measures for clinical trials. Future research in the context of international collaborations on agreed gene sets/classifiers (e.g., Banff Working Group for Molecular Pathology) should assess non-selective populations of transplant biopsies and determine clinically meaningful molecular scoring systems that predict cAMR development and graft failure. These studies should include multivariate analyses in combination with traditional clinical, histopathological, or immunogenetic parameters.

### Subclinical AMR Including Incomplete Phenotypes

Table 9 lists publications that describe subclinical AMR in protocol biopsies, including incomplete phenotypes, and all studies linking Banff diagnostic categories and subcategories to outcomes in protocol biopsies (34, 80, 94–99). Subclinical AMR diagnosed in protocol biopsies is associated with subsequent chronic kidney injury, impaired graft function, and impaired graft survival, but whether treatment of subclinical AMR diagnosed in protocol biopsies improves graft outcomes is not proven. The quality of evidence is not high.

**TABLE 9:** Studies investigating outcomes in cases with subclinical AMR, including incomplete phenotypes (34, 80, 94–99).

References	Endpoint	Definition of predictor	Findings	Level of evidence (grade)
Loupy et al. (34)	GFR, TG, IFTA	Subclinical AMR	Patients with subclinical AMR at 3 mo had at 1 year: Higher rate of IFTA (100% vs. 33.3%; *p* < 0.01) Higher rate of TG (43% vs. 0%; *p* = 0.02) Lower mGFR (39.2 ± 13.9 vs. 61.9 ± 19.2 ml/min/1.73 m^2^; *p* < 0.01)	Low
Lerut et al. (94)	PTCML, cAMR	PTC	Protocol biopsies with ptc at 3 mo associated with PTCML (*p* < 0.0001)/cAMR (*p* = 0.0002) at 1 year	Low
Haas et al. (95)	CAN score (cg + ci + ct + cv)	Subclinical AMR	Subclinical AMR (stable SCr, PTC, diffuse PTC C4d, positive DSA) during 1st year post transplantation associated with higher increase in CAN score in follow-up biopsies 335 ± 248 (SD) days later (3.5 ± 2.5 vs. 1.0 ± 2.0; *p* = 0.01)	Low
Loupy et al. (96)	cAMR	MVI + class II DSA	Multivariate analysis demonstrated that presence of MVI and anti-HLA class II DSA at 3 mo was associated with a 4-fold increased risk of progression to cAMR independently of C4d (*p* < 0.05)	Low
Cosio et al. (97)	Graft loss	‘cAMR’ (= cg > 0 or MVI≥2)	4.2% of protocol biopsies at 1 year showed cAMR; risk of death-censored graft survival HR 12.6 (95% CI 6.58–24.3; *p* < 0.0001)	Moderate
Gloor et al. (80)	GFR, proteinuria	TG	Prognosis of subclinical TG was equally poor as TG diagnosed with graft dysfunction, with progressive worsening of histopathologic changes and function	Low
Papadimitriou et al. (98)		NR	Indication + protocol biopsies (concurrent DSA): More incomplete phenotype in protocol than in indication biopsies Persistence/worsening of AMR in a subsequent biopsy occurred in 38.2% of cases independently of strength of AMR findings in 1st biopsy (e.g., progression to cAMR occurred also in cases with suspicious or non-diagnostic findings)	Low
Tsuji et al. (99)	cAMR	MVI	MVI in protocol biopsies at 3 mo correlates with later development of cAMR (*p* = 0.03)	Low

AMR, antibody-mediated rejection; c, chronic; DSA, donor-specific antibody; GFR, glomerular filtration rate; HLA, human leukocyte antigen; IFTA, interstitial fibrosis and tubular atrophy; m, mean; MVI, microvascular inflammation; NR, not reported; PTC, peritubular capillary; PTCML, peritubular capillary basement membrane multilayering; sCr, serum creatinine; SD, standard deviation; TG, transplant glomerulopathy.

A literature search for studies evaluating the frequency of subclinical AMR management showed that ∼60% of patients received treatment, usually with antibody-targeted therapies. Again, national variations were observed. In Paris, 57% of patients with subclinical AMR received antirejection therapy (36) while US centers treated subclinical AMR more aggressively than elsewhere (100); centers in Canada (101) and Belgium (59) treated this presentation very selectively. Differences may also relate to whether centers perform high-risk transplantations and the timing of the post-transplant biopsy. Early (e.g., 1- or 3-month) post-transplant subclinical AMR in patients at high immunological risk may have different outcomes than late (e.g., ≥1-year) post-transplant subclinical AMR in patients with *dn*DSA. Given that subclinical AMR in protocol biopsies appears to be associated with impaired graft survival, but protocol biopsies are not universally performed, and the management of subclinical AMR is heterogeneous, it is unsurprising that consensus documents do not provide guidance (28, 102). We consider that identifying AMR in protocol biopsies could be a clinically meaningful endpoint as an independent predictor of graft loss; but in the absence of high-quality evidence and uncertainty about the effect of treatment, we remain cautious. The priority should be to agree good definitions for the phenotypes and endpoints of AMR that are clinically meaningful in kidney transplantation studies. We also recommend that further research investigates the role of subclinical AMR in graft failure.

### Restricted Definition of Banff Classification of AMR for Use as Endpoint

Based on the evidence presented above, we propose a restricted definition of the Banff phenotypes of AMR, if used as an endpoint in interventional trials (Table 10) (19, 22).

**TABLE 10:** Restricting the Banff classification for AMR for the purpose of endpoints in clinical trials, based on the evidence reviewed (19, 22).

Banff 2017 Category 2: Antibody-mediated changes	Restricted definition of AMR for use as primary endpoint
**Active AMR; all criteria must be met for diagnosis**	**Active AMR; all criteria must be met for diagnosis**
1. Histologic evidence of acute tissue injury, including ≥1 of the following	1. Histologic evidence of acute tissue injury
• MVI (g > 0 and/or ptc>0), in the absence of recurrent or *de novo* glomerulonephritis, although in the presence of aTCMR, borderline infiltrate, or infection, ptc≥1 alone is not sufficient and g must be ≥ 1	• At least moderate MVI (g + ptc≥2 with g ≥ 1 in the presence of aTCMR, caTCMR, or borderline changes, or infectious disease of the transplant)
• Intimal or transmural arteritis (v > 0)	• —
• Acute TMA the absence of any other cause	• —
• Acute tubular injury, in the absence of any other apparent cause	• —
2. Evidence of current/recent antibody interaction with vascular endothelium, including one or more of the following	2. Evidence of current/recent antibody interaction with vascular endothelium
• Linear C4d staining in peritubular capillaries (C4d2 or C4d3 by IF on frozen sections, or C4d > 0 by IHC on paraffin-embedded sections)	• At least MVI g + ptc≥2 with g ≥ 1 in the presence of aTCMR, caTCMR or borderline changes, or infectious disease of the transplant), identical to criterion 1 for aAMR
• At least moderate MVI ([g + ptc]≥2) in absence of recurrent/*dn* glomerulonephritis, although in the presence of aTCMR, borderline infiltrate, or infection, ptc≥2 alone is not sufficient and g must be ≥ 1	• With or without C4d positivity (C4d ≥ 1 on paraffin tissue or ≥2 on frozen tissue)
• Increased expression of gene transcripts/classifiers in the biopsy tissue strongly associated with AMR, if thoroughly validated	• —
3. Serologic evidence of donor-specific antibodies (DSA to HLA or other antigens). C4d staining or expression of validated transcripts/classifiers as noted above in criterion 2 may substitute for DSA; however thorough DSA testing, including testing for non-HLA antibodies if HLA antibody testing is negative, is strongly advised whenever criteria 1 and 2 are met	3. DSA positivity (anti-HLA antibodies)
**Chronic active AMR; all criteria must be met for diagnosis**	**Chronic active AMR; all criteria must be met for diagnosis**
1. Morphologic evidence of chronic tissue injury, one or more of the following	1. Morphologic evidence of chronic tissue injury, including ≥1 of the following
• TGA (cg > 0) if no evidence of cTMA or chronic recurrent/*dn* glomerulonephritis; includes changes evident by EM alone (cg1a)	• cg ≥ 1 according to Banff 2011 (≥10% of the glomerular capillary walls in the most severely affected glomerulus involved)
• Severe peritubular capillary basement membrane multilayering (requires EM)	• —
• Arterial intimal fibrosis of new onset, excluding other causes; leukocytes within the sclerotic intima favor cAMR if there is no prior history of TCMR, but are not required	• —
2. Identical to criterion 2 for aAMR, above	2. Identical to criterion 2 for aAMR, above
3. Identical to criterion 3 for aAMR, above, including strong recommendation for DSA testing whenever criteria 1 and 2 are met	3. DSA positivity (anti-HLA antibodies)
**Chronic AMR; all criteria must be met for diagnosis**	**Chronic AMR; all criteria must be met for diagnosis**
1. Morphologic evidence of chronic tissue injury, including ≥1 of the following	1. Morphologic evidence of chronic tissue injury
• Transplant glomerulopathy (cg > 0) if no evidence of cTMA or chronic recurrent/*dn* glomerulonephritis; includes changes evident by EM alone (cg1a)	• Cg ≥ 1 according to Banff 2011 (≥10% of the glomerular capillary walls in the most severely affected glomerulus involved) if no evidence of TMA of any other cause or recurrent or *dn* glomerulopathy
• Severe PTCML (requires EM)	• —
2. Absence of evidence of current/recent antibody interaction with the endothelium (criterion 2 for active AMR, above)	2. Absence of evidence of current/recent antibody interaction with the endothelium (criterion 2 for aAMR, above)
3. Prior documented diagnosis of a or caAMR or documented prior evidence of DSA	3. Prior documented diagnosis of a or caAMR or documented prior evidence of DSA

a, acute/active; AMR, antibody-mediated rejection; ca, chronic active; *dn*, *de novo*; DSA, donor-specific antibody; EM, electron microscopy; HLA, human leukocyte antigen; IF, immunofluorescence; IHC, immunohistochemistry; MVI, microvascular inflammation; PTCML, peritubular capillary basement membrane multilayering; TCMR, T cell-mediated rejection; TMA, thrombotic microangiopathy.

## Conclusions

• Evidence relating to the relationship between AMR and outcomes is largely based on retrospective analyses that do not utilize the strict, most recent Banff categories of AMR, but instead investigate individual features of AMR, combinations of individual features of AMR, or combined Banff categories (such as combining aAMR and caAMR).
  ○ Strongest evidence for associations between individual features and impaired graft outcome is noted for MVI score ≥2 (if borderline changes, aTCMR or infection are present, g + ptc>1 is not sufficient and g > 1 is required) and cg>10% (>10% of the most severely affected glomerulus).
  ○ Together with presence of HLA-DSA, these parameters should be the basis for AMR endpoints, acknowledging their limitations (lack of specificity, between-study heterogeneity in definitions used, and high interobserver variability).
• Based on evidence for association between individual features of AMR and outcome, AMR diagnosed in indication or protocol biopsies should be considered as a primary endpoint in clinical trials for kidney transplantation.
• However, based on available evidence, we suggest refinement of the Banff 2017 definition for AMR diagnosis to the following three AMR-related endpoints:
  ○ Restricted aAMR, defined by the conservative threshold of at least moderate MVI (g + ptc≥2 with g ≥ 1 in the presence of aTCMR, caTCMR, or borderline changes) and DSA positivity (anti-HLA antibodies) with or without C4d positivity (C4d ≥ 1 on paraffin tissue or ≥2 on frozen tissue).
  ○ Restricted caAMR, defined by the conservative threshold of cg ≥ 1 according to Banff 2011 (≥10% of the glomerular capillary walls in the most severely affected glomerulus involved) plus at least moderate MVI (g + ptc≥2 with g ≥ 1 in the presence of aTCMR, caTCMR, or borderline changes) and DSA positivity (anti-HLA antibodies) with or without C4d positivity (C4d ≥ 1 on paraffin-embedded tissue; ≥2 on frozen tissue).
  ○ Restricted cAMR, defined by the conservative threshold of cg ≥ 1 according to Banff 2011 (≥10% of glomerular capillary walls in most severely affected glomerulus involved) and current or past DSA positivity (anti-HLA antibodies) with or without C4d positivity (C4d ≥ 1 on paraffin-embedded tissue; ≥2 on frozen tissue).
• Other features of AMR used in Banff AMR definitions (ATI in the absence of any other cause; TMA; Banff Lesion Score v ≥ 1; increased transcripts associated with AMR; cg<10%; PTCML; arterial intimal fibrosis of new onset; DSA− cases) show less-robust evidence than MVI score ≥2 and cg>10%.
  ○ In isolation, without the other features of AMR described above, these features should not be considered as efficacy endpoints for clinical trials.
• The use of histology as endpoint for studies after kidney transplantation needs to consider that histological scoring reproducibility is at best moderate.
• There is a clear need for additional investigations of outcomes for all features and all categories of AMR.
  ○ Any such studies should follow the Banff 2017 recommendations on best practice for pathology endpoints in clinical trials (19), in particular involving pathologists in clinical trial design, use of a panel of pathologists for grading with a defined adjudication mechanism, granular scoring and reporting of histological data as continuous parameters and, where possible, maintaining a digital archive of pathology slides to facilitate external validation; use of data lumped into arbitrarily defined ‘AMR’ is discouraged.

### Scientific Advice from the Committee for Medicinal Products for Human Use (CHMP) of the European Medicines Agency (EMA) Regarding These Conclusions

• The CHMP recognized the issues in defining AMR.
• The CHMP welcomed and endorsed the suggestion to initiate a discussion on the use of the Banff classification as a tool to define AMR as an endpoint in clinical trials, in addition to its diagnostic and research value.
• The rationale behind the restricted definitions of aAMR and caAMR for use as primary endpoints was well received by the CHMP.
  ○ For this to happen, evidence-based classification and state-of-the-art, transparent, and standardized review processes of scientific data are required to demonstrate the usefulness of the restricted Banff definitions for AMR.

## References

[1] European Medicines Agency. Clinical Investigation of Immunosuppressants for Solid Organ Transplantation (2008). CHMP/EWP/263148/06. Available at: https://www.ema.europa.eu/en/clinical-investigation-immunosuppressants-solid-organ-transplantation (Accessed October 18, 2021).

[2] Racusen LC Solez K Colvin RB Bonsib SM Castro MC Cavallo T The Banff 97 Working Classification of Renal Allograft Pathology. Kidney Int (1999) 55:713–23. 10.1046/j.1523-1755.1999.00299.x 9987096

[3] Naesens M Kuypers DRJ De Vusser K Evenepoel P Claes K Bammens B The Histology of Kidney Transplant Failure. Transplantation (2014) 98:427–35. 10.1097/tp.0000000000000183 25243513

[4] Sellarés J De Freitas DG Mengel M Reeve J Einecke G Sis B Understanding the Causes of Kidney Transplant Failure: the Dominant Role of Antibody-Mediated Rejection and Nonadherence. Am J Transplant (2012) 12:388–99. 10.1111/j.1600-6143.2011.03840.x 22081892

[5] El-Zoghby ZM Stegall MD Lager DJ Kremers WK Amer H Gloor JM Identifying Specific Causes of Kidney Allograft Loss. Am J Transplant (2009) 9:527–35. 10.1111/j.1600-6143.2008.02519.x 19191769

[6] Chand S Atkinson D Collins C Briggs D Ball S Sharif A The Spectrum of Renal Allograft Failure. PLoS One (2016) 11:e0162278. 10.1371/journal.pone.0162278 27649571 PMC5029903

[7] Mayrdorfer M Liefeldt L Wu K Rudolph B Zhang Q Friedersdorff F Exploring the Complexity of Death-Censored Kidney Allograft Failure. J Am Soc Nephrol (2021) 32:1513–26. 10.1681/asn.2020081215 33883251 PMC8259637

[8] Chehade H Pascual M. The challenge of Acute Antibody-Mediated Rejection in Kidney Transplantation. Transplantation (2016) 100:264–5. 10.1097/tp.0000000000000959 26479286

[9] Wehmeier C Amico P Hirt-Minkowski P Georgalis A Höenger G Menter T Acute Rejection Phenotypes in the Current Era of Immunosuppression: a Single-center Analysis. Transplant Direct (2017) 3:e136. 10.1097/txd.0000000000000650 28361120 PMC5367753

[10] Loupy A Lefaucheur C. Antibody-mediated Rejection of Solid-Organ Allografts. N Engl J Med (2018) 379:1150–60. 10.1056/nejmra1802677 30231232

[11] Karahan GE Claas FHJ Heidt S. Technical Challenges and Clinical Relevance of Single Antigen Bead C1q/C3d Testing and IgG Subclass Analysis of Human Leukocyte Antigen Antibodies. Transpl Int (2018) 31:1189–97. 10.1111/tri.13327 30091231

[12] Roelen DL Doxiadis II Claas FHJ. Detection and Clinical Relevance of Donor Specific HLA Antibodies: a Matter of Debate. Transpl Int (2012) 25:604–10. 10.1111/j.1432-2277.2012.01491.x 22587521

[13] Reindl-Schwaighofer R Heinzel A Gualdoni GA Mesnard L Claas FHJ Oberbauer R. Novel Insights into Non-HLA Alloimmunity in Kidney Transplantation. Transpl Int (2020) 33:5–17. 10.1111/tri.13546 PMC6972536 31650645

[14] Patel R Terasaki PI. Significance of the Positive Crossmatch Test in Kidney Transplantation. N Engl J Med (1969) 280:735–9. 10.1056/nejm196904032801401 4886455

[15] Solez Ki. m. Axelsen RA Benediktsson H Burdick JF Cohen AH Colvin RB International Standardization of Criteria for the Histologic Diagnosis of Renal Allograft Rejection: the Banff Working Classification of Kidney Transplant Pathology. Kidney Int (1993) 44:411–22. 10.1038/ki.1993.259 8377384

[16] Regele H Exner M Watschinger B Wenter C Wahrmann M Österreicher C Endothelial C4d Deposition Is Associated with Inferior Kidney Allograft Outcome Independently of Cellular Rejection. Nephrol Dial Transplant (2001) 16:2058–66. 10.1093/ndt/16.10.2058 11572897

[17] Racusen LC Colvin RB Solez K Mihatsch MJ Halloran PF Campbell PM Antibody-Mediated Rejection Criteria - an Addition to the Banff ′97 Classification of Renal Allograft Rejection. Am J Transplant (2003) 3:708–14. 10.1034/j.1600-6143.2003.00072.x 12780562

[18] Solez K Colvin RB Racusen LC Haas M Sis B Mengel M Banff 07 Classification of Renal Allograft Pathology: Updates and Future Directions. Am J Transplant (2008) 8:753–60. 10.1111/j.1600-6143.2008.02159.x 18294345

[19] Haas M Loupy A Lefaucheur C Roufosse C Glotz D Seron D The Banff 2017 Kidney Meeting Report: Revised Diagnostic Criteria for Chronic Active T Cell-Mediated Rejection, Antibody‐mediated Rejection, and Prospects for Integrative Endpoints for Next‐generation Clinical Trials. Am J Transplant (2018) 18:293–307. 10.1111/ajt.14625 29243394 PMC5817248

[20] Sis B Mengel M Haas M Colvin RB Halloran PF Racusen LC Banff '09 Meeting Report: Antibody Mediated Graft Deterioration and Implementation of Banff Working Groups. Am J Transplant (2010) 10:464–71. 10.1111/j.1600-6143.2009.02987.x 20121738

[21] Haas M Sis B Racusen LC Solez K Glotz D Colvin RB Banff 2013 Meeting Report: Inclusion of C4d-Negative Antibody-Mediated Rejection and Antibody-Associated Arterial Lesions. Am J Transplant (2014) 14:272–83. 10.1111/ajt.12590 24472190

[22] Loupy A Haas M Roufosse C Naesens M Adam B Afrouzian M The Banff 2019 Kidney Meeting Report (I): Updates on and Clarification of Criteria for T Cell- and Antibody‐mediated Rejection. Am J Transplant (2020) 20:2318–31. 10.1111/ajt.15898 32463180 PMC7496245

[23] Roufosse C Simmonds N Clahsen-van Groningen M Haas M Henriksen KJ Horsfield C A 2018 Reference Guide to the Banff Classification of Renal Allograft Pathology. Transplantation (2018) 102:1795–814. 10.1097/tp.0000000000002366 30028786 PMC7597974

[24] Smith B Cornell LD Smith M Cortese C Geiger X Alexander MP A Method to Reduce Variability in Scoring Antibody-Mediated Rejection in Renal Allografts: Implications for Clinical Trials - a Retrospective Study. Transpl Int (2018) 32:173–83. 10.1111/tri.13340 30179275 PMC6328317

[25] Preka E Sekar T Lopez Garcia SC Shaw O Kessaris N Mamode N Outcomes of Paediatric Kidney Transplant Recipients Using the Updated 2013/2017 Banff Histopathological Classification for Antibody-Mediated Rejection. Pediatr Nephrol (2021) 36:2575–85. 10.1007/s00467-021-05103-x 34143297

[26] De Serres SA Noël R Côté I Lapointe I Wagner E Riopel J Banff Criteria for Chronic Active Antibody-Mediated Rejection: Assessment in a Real-Life Setting. Am J Transplant (2013) 16:1516–25. 10.1111/ajt.13624 26602055

[27] De Kort H Moran L Roufosse C. The Role of Electron Microscopy in Renal Allograft Biopsy Evaluation. Curr Opin Organ Transpl (2015) 20:333–42. 10.1097/mot.0000000000000183 25944229

[28] Schinstock CA Mannon RB Budde K Chong AS Haas M Knechtle S Recommended Treatment for Antibody-Mediated Rejection after Kidney Transplantation: The 2019 Expert Consensus from the Transplantion Society Working Group. Transplantation (2020) 104:911–22. 10.1097/tp.0000000000003095 31895348 PMC7176344

[29] Solar-Cafaggi D Marino L Uribe-Uribe N Morales-Buenrostro LE. Antibody-mediated Rejection in the Banff Classifications of 2007 and 2017: a Comparison of Renal Graft Loss Prediction Capability. Transpl Immunol (2018) 51:40–4. 10.1016/j.trim.2018.08.008 30170180

[30] Sai K OmotoShimizu KT Shimizu T Honda K Tanabe K. The Impact of C4d-Negative Acute Antibody-Mediated Rejection on Short-Term Prognosis Among Kidney Transplant Recipients. Nephrology (2015) 20(Suppl. 2):16–9. 10.1111/nep.12473 26031580

[31] Orandi BJ Chow EHK Hsu A Gupta N Van Arendonk KJ Garonzik-Wang JM Quantifying Renal Allograft Loss Following Early Antibody-Mediated Rejection. Am J Transplant (2015) 15:489–98. 10.1111/ajt.12982 25611786 PMC4304875

[32] Orandi BJ Alachkar N Kraus ES Naqvi F Lonze BE Lees L Presentation and Outcomes of C4d‐Negative Antibody‐Mediated Rejection after Kidney Transplantation. Am J Transplant (2016) 16:213–20. 10.1111/ajt.13434 26317487 PMC6114097

[33] Everly MJ Everly JJ Arend LJ Brailey P Susskind B Govil A Reducing De Novo Donor-specific Antibody Levels during Acute Rejection Diminishes Renal Allograft Loss. Am J Transplant (2009) 9:1063–71. 10.1111/j.1600-6143.2009.02577.x 19344434

[34] Loupy A Suberbielle-Boissel C Hill GS Lefaucheur C Anglicheau D Zuber J Outcome of Subclinical Antibody-Mediated Rejection in Kidney Transplant Recipients with Preformed Donor-specific Antibodies. Am J Transplant (2009) 9:2561–70. 10.1111/j.1600-6143.2009.02813.x 19775320

[35] Wu K Budde K Schmidt D Neumayer H-H Rudolph B. The Relationship of the Severity and Category of Acute Rejection with Intimal Arteritis Defined in Banff Classification to Clinical Outcomes. Transplantation (2015) 99:e105–e114. 10.1097/tp.0000000000000640 25719260

[36] Loupy A Vernerey D Tinel C Aubert O Duong van Huyen J-P Rabant M Subclinical Rejection Phenotypes at 1 Year post-transplant and Outcome of Kidney Allografts. J Am Soc Nephrol (2015) 26:1721–31. 10.1681/asn.2014040399 25556173 PMC4483584

[37] Lesage J Noël R Lapointe I Côté I Wagner E Désy O Donor-specific Antibodies, C4d and Their Relationship with the Prognosis of Transplant Glomerulopathy. Transplantation (2015) 99:69–76. 10.1097/tp.0000000000000310 25073036

[38] Wavamunno MD O'Connell PJ Vitalone M Fung CL-S Allen RDM Chapman JR Transplant Glomerulopathy: Ultrastructural Abnormalities Occur Early in Longitudinal Analysis of Protocol Biopsies. Am J Transplant (2007) 7:2757–68. 10.1111/j.1600-6143.2007.01995.x 17924997

[39] Perkowska-Ptasińska A Ciszek M Chmura A Galazka Z Paczek L Durlik M Transplant Glomerulopathy: Clinical and Pathological Correlations. Transplant Proc (2009) 41:141–9. 10.1016/j.transproceed.2008.10.052 19249499

[40] Shimizu T Ishida H Toki D Nozaki T Omoto K Tanabe K Clinical and Pathological Analyses of Transplant Glomerulopathy Cases. Nephrology (2014) 19:21–6. 10.1111/nep.12243 24842817

[41] Hayde N Bao Y Pullman J Ye B Calder RB Chung M The Clinical and Genomic Significance of Donor-specific Antibody-positive/C4d-Negative and Donor-specific Antibody-negative/C4d-Negative Transplant Glomerulopathy. Clin J Am Soc Nephrol (2013) 8:2141–8. 10.2215/cjn.04240413 24030736 PMC3848403

[42] Péfaur J Díaz P Panace R Salinas P Fiabane A Quinteros N Early and Late Humoral Rejection: a Clinicopathologic Entity in Two Times. Transplant Proc (2008) 40:3229–36. 10.1016/j.transproceed.2008.03.123 19010241

[43] Shimizu T Ishida H Shirakawa H Omoto K Tsunoyama K Iida S Clinicopathological Analysis of Transplant Glomerulopathy Cases. Clin Transplant (2009) 23:39–43. 10.1111/j.1399-0012.2009.01008.x 19594595

[44] John R Konvalinka A Tobar A Kim SJ Reich HN Herzenberg AM. Determinants of Long-Term Graft Outcome in Transplant Glomerulopathy. Transplantation (2010) 90:757–64. 10.1097/tp.0b013e3181efcffd 20838279

[45] Nair R Fraer M Agrawal N Suneja M. Acute Transplant Glomerulopathy Is Associated with Antibody-Mediated Rejection and Poor Graft Outcome. Transplant Proc (2010) 42:3507–12. 10.1016/j.transproceed.2010.06.020 21094805

[46] López Jiménez V Fuentes L Jiménez T León M Garcia I Sola E Transplant Glomerulopathy: Clinical Course and Factors Relating to Graft Survival. Transplant Proc (2012) 44:2599–600. 10.1016/j.transproceed.2012.09.068 23146467

[47] Kamal L Broin PÓ Bao Y Ajaimy M Lubetzky M Gupta A Clinical, Histological, and Molecular Markers Associated with Allograft Loss in Transplant Glomerulopathy Patients. Transplantation (2015) 99:1912–8. 10.1097/tp.0000000000000598 25675205

[48] Dobi D Bodó Z Kemény É Bidiga L Hódi Z Szenohradszky P Peritubular Capillary Basement Membrane Multilayering in Early and Advanced Transplant Glomerulopathy: Quantitative Parameters and Diagnostic Aspects. Virchows Arch (2016) 469:563–73. 10.1007/s00428-016-2010-1 27605054

[49] Halloran PF Merino Lopez M Barreto Pereira A. Identifying Subphenotypes of Antibody-Mediated Rejection in Kidney Transplants. Am J Transplant (2016) 16:908–20. 10.1111/ajt.13551 26743766

[50] Toki D Inui M Ishida H Okumi M Shimizu T Shirakawa H Interstitial Fibrosis Is the Critical Determinant of Impaired Renal Function in Transplant Glomerulopathy. Nephrology (2016) 21(Suppl. 1):20–5. 10.1111/nep.12765 26970313

[51] Courant M Visentin J Linares G Dubois V Lepreux S Guidicelli G The Disappointing Contribution of Anti-human Leukocyte Antigen Donor-specific Antibodies Characteristics for Predicting Allograft Loss. Nephrol Dial Transplant (2018) 33:1853–63. 10.1093/ndt/gfy088 29672702

[52] Lubetzky M Hayde N Ó Broin P Ajaimy M Bao Y Mohammed O Molecular Signatures and Clinical Outcomes of Transplant Glomerulopathy Stratified by Microvascular Inflammation and Donor-specific Antibody. Clin Transplant (2018) 33:e13469. 10.1111/ctr.13469 30578675

[53] Sablik KA Clahsen-Van Groningen MC Looman CWN Damman J Roelen DL van Agteren M Chronic-active Antibody-Mediated Rejection with or without Donor-specific Antibodies Has Similar Histomorphology and Clinical Outcome - a Retrospective Study. Transpl Int (2018) 31:900–8. 10.1111/tri.13154 29570868

[54] Aubert O Higgins S Bouatou Y Yoo D Raynaud M Viglietti D Archetype Analysis Identifies Distinct Profiles in Renal Transplant Recipients with Transplant Glomerulopathy Associated with Allograft Survival. J Am Soc Nephrol (2019) 30:625–39. 10.1681/asn.2018070777 30872323 PMC6442337

[55] Mengel M Sis B Haas M Colvin RB Halloran PF Racusen LC Banff 2011 Meeting Report: New Concepts in Antibody-Mediated Rejection. Am J Transplant (2012) 12:563–70. 10.1111/j.1600-6143.2011.03926.x 22300494 PMC3728651

[56] Senev A Van Loon E Lerut E Callemeyn J Coemans M Van Sandt V Risk Factors, Histopathological Features, and Graft Outcome of Transplant Glomerulopathy in the Absence of Donor-specific HLA Antibodies. Kidney Int (2021) 100:401–14. 10.1016/j.kint.2021.01.029 33675843

[57] Zhang Q Rudolph B Choi M Bachmann F Schmidt D Duerr M The Relationship between Proteinuria and Allograft Survival in Patients with Transplant Glomerulopathy: a Retrospective Single‐center Cohort Study. Transpl Int (2021) 34:259–71. 10.1111/tri.13787 33205460

[58] Kovács G Devercelli G Zelei T Hirji I Vokó Z Keown PA. Association between Transplant Glomerulopathy and Graft Outcomes Following Kidney Transplantation: A Meta-Analysis. PLoS One (2020) 15:e0231646. 10.1371/journal.pone.0231646 32343692 PMC7188300

[59] Senev A Coemans M Lerut E Van Sandt V Daniëls L Kuypers D Histological Picture of Antibody‐mediated Rejection without Donor‐specific anti‐HLA Antibodies: Clinical Presentation and Implications for Outcome. Am J Transplant (2019) 19:763–80. 10.1111/ajt.15074 30107078

[60] Mauiyyedi S Crespo M Collins AB Schneeberger EE Pascual MA Saidman SL Acute Humoral Rejection in Kidney Transplantation: II. Morphology, Immunopathology, and Pathologic Classification. J Am Soc Nephrol (2002) 13:779–87. 10.1681/asn.v133779 11856785

[61] Haas M Ratner LE Montgomery RA. C4d Staining of Perioperative Renal Transplant Biopsies1. Transplantation (2002) 74:711–7. 10.1097/00007890-200209150-00021 12352891

[62] Djamali A Muth BL Ellis TM Mohamed M Fernandez LA Miller KM Increased C4d in post-reperfusion Biopsies and Increased Donor Specific Antibodies at One-Week post Transplant Are Risk Factors for Acute Rejection in Mild to Moderately Sensitized Kidney Transplant Recipients. Kidney Int (2013) 83:1185–92. 10.1038/ki.2013.44 23447068 PMC3672254

[63] Kikić Ž Kainz A Kozakowski N Oberbauer R Regele H Bond G Capillary C4d and Kidney Allograft Outcome in Relation to Morphologic Lesions Suggestive of Antibody-Mediated Rejection. Clin J Am Soc Nephrol (2015) 10:1435–43. 10.2215/CJN.09901014 26071493 PMC4527028

[64] Nickeleit V Zeiler M Gudat F Thiel G Mihatsch MJ. Detection of the Complement Degradation Product C4d in Renal Allografts: Diagnostic and Therapeutic Implications. J Am Soc Nephrol (2002) 13:242–51. 10.1681/asn.v131242 11752044

[65] Dickenmann M Steiger J Descœudres B Mihatsch M Nickeleit V. The Fate of C4d Positive Kidney Allografts Lacking Histological Signs of Acute Rejection. Clin Nephrol (2006) 65:173–9. 10.5414/cnp65173 16550748

[66] Dominy KM Willicombe M Al Johani T Beckwith H Goodall D Brookes P Molecular Assessment of C4d-Positive Renal Transplant Biopsies without Evidence of Rejection. Kidney Int Rep (2019) 4:148–58. 10.1016/j.ekir.2018.09.005 30596178 PMC6308373

[67] Lefaucheur C Loupy A Vernerey D Duong-Van-Huyen J-P Suberbielle C Anglicheau D Antibody-mediated Vascular Rejection of Kidney Allografts: a Population-Based Study. Lancet (2013) 381:313–9. 10.1016/s0140-6736(12)61265-3 23182298

[68] Loupy A Haas M Solez K Racusen L Glotz D Seron D The Banff 2015 Kidney Meeting Report: Current Challenges in Rejection Classification and Prospects for Adopting Molecular Pathology. Am J Transplant (2017) 17:28–41. 10.1111/ajt.14107 27862883 PMC5363228

[69] Le Quintrec M Lionet A Kamar N Karras A Barbier S Buchler M Complement Mutation-AssociatedDe NovoThrombotic Microangiopathy Following Kidney Transplantation. Am J Transplant (20082008) 8:1694–701. 10.1111/j.1600-6143.2008.02297.x 18557729

[70] Satoskar AA Pelletier R Adams P Nadasdy GM Brodsky S Pesavento T De Novo thrombotic Microangiopathy in Renal Allograft Biopsies-Role of Antibody-Mediated Rejection. Am J Transplant (2010) 10:1804–11. 10.1111/j.1600-6143.2010.03178.x 20659088

[71] Chua JS Baelde HJ Zandbergen M Wilhelmus S van Es LA de Fijter JW Complement Factor C4d Is a Common Denominator in Thrombotic Microangiopathy. J Am Soc Nephrol (2015) 26:2239–47. 10.1681/asn.2014050429 25573909 PMC4552108

[72] Haas M Mirocha J. Early Ultrastructural Changes in Renal Allografts: Correlation with Antibody-Mediated Rejection and Transplant Glomerulopathy. Am J Transplant (2011) 11:2123–31. 10.1111/j.1600-6143.2011.03647.x 21827618

[73] Bagnasco SM Zachary AA Racusen LC Arend LJ Carter-Monroe N Alachkar N Time Course of Pathologic Changes in Kidney Allografts of Positive Crossmatch HLA-Incompatible Transplant Recipients. Transplantation (2014) 97:440–5. 10.1097/01.tp.0000437177.40551.f4 24531821

[74] Einecke G Sis B Reeve J Mengel M Campbell PM Hidalgo LG Antibody-mediated Microcirculation Injury Is the Major Cause of Late Kidney Transplant Failure. Am J Transplant (2009) 9:2520–31. 10.1111/j.1600-6143.2009.02799.x 19843030

[75] Sis B Jhangri GS Riopel J Chang J de Freitas DG Hidalgo L A New Diagnostic Algorithm for Antibody-Mediated Microcirculation Inflammation in Kidney Transplants. Am J Transplant (2012) 12:1168–79. 10.1111/j.1600-6143.2011.03931.x 22300601

[76] Verghese P Dunn T Najafian B Kim Y Matas A. The Impact of C4d and Microvascular Inflammation before We Knew Them. Clin Transplant (2013) 27:388–96. 10.1111/ctr.12111 23528049

[77] De Kort H Willicombe M Brookes P Dominy KM Santos-Nunez E Galliford JW Microcirculation Inflammation Associates with Outcome in Renal Transplant Patients WithDe NovoDonor-specific Antibodies. Am J Transplant (2013) 13:485–92. 10.1111/j.1600-6143.2012.04325.x 23167441

[78] Sapir-Pichhadze R Curran SP John R Tricco AC Uleryk E Laupacis A A Systematic Review of the Role of C4d in the Diagnosis of Acute Antibody-Mediated Rejection. Kidney Int (2015) 87:182–94. 10.1038/ki.2014.166 24827778

[79] Matas AJ Fieberg A Mannon RB Leduc R Grande J Kasiske BL Long‐term Follow‐up of the DeKAF Cross‐sectional Cohort Study. Am J Transplant (2019) 19:1432–43. 10.1111/ajt.15204 30506642 PMC7653899

[80] Gloor JM Sethi S Stegall MD Park WD Moore SB DeGoey S Transplant Glomerulopathy: Subclinical Incidence and Association with Alloantibody. Am J Transplant (2007) 7:2124–32. 10.1111/j.1600-6143.2007.01895.x 17608832

[81] Torres IB Salcedo M Moreso F Sellarés J Castellá E Azancot MA Comparing Transplant Glomerulopathy in the Absence of C4d Deposition and Donor-specific Antibodies to Chronic Antibody-Mediated Rejection. Clin Transplant (2014) 28:1148–54. 10.1111/ctr.12433 25103874

[82] Baid-Agrawal S Farris AB 3rd Pascual M Mauiyyedi S Farrell ML Tolkoff-Rubin N Overlapping Pathways to Transplant Glomerulopathy: Chronic Humoral Rejection, Hepatitis C Infection, and Thrombotic Microangiopathy. Kidney Int (2011) 80:879–85. 10.1038/ki.2011.194 21697808

[83] Olson JL. Renal Disease Caused by Hypertension. In: Jennette JC, editor. Heptinstall’s Pathology of the Kidney. 7th ed. Philadelphia, PA: Wolters Kluwer (2015).

[84] Einecke G Reeve J Sis B Mengel M Hidalgo L Famulski KS A Molecular Classifier for Predicting Future Graft Loss in Late Kidney Transplant Biopsies. J Clin Invest (2010) 120:1862–72. 10.1172/jci41789 20501945 PMC2877953

[85] Roufosse CA Shore I Moss J Moran LB Willicombe M Galliford J Peritubular Capillary Basement Membrane Multilayering on Electron Microscopy. Transplantation (2012) 94:269–74. 10.1097/tp.0b013e31825774ab 22790448

[86] De Kort H Willicombe M Brookes P Moran LB Santos-Nunez E Galliford JW Peritubular Capillary Basement Membrane Multilayering in Renal Allograft Biopsies of Patients with De Novo Donor-specific Antibodies. Transplantation (2016) 100:889–97. 10.1097/tp.0000000000000908 26413993

[87] Sijpkens YW Doxiadis II Van Kemenade FJ Zwinderman AH De Fijter JW Claas FH Chronic Rejection with or without Transplant Vasculopathy. Clin Transplant (2003) 17:163–70. 10.1034/j.1399-0012.2003.00039.x 12780663

[88] Loupy A Lefaucheur C Vernerey D Chang J Hidalgo LG Beuscart T Molecular Microscope Strategy to Improve Risk Stratification in Early Antibody-Mediated Kidney Allograft Rejection. J Am Soc Nephrol (2014) 25:2267–77. 10.1681/asn.2013111149 24700874 PMC4178445

[89] Yazdani S Callemeyn J Gazut S Lerut E de Loor H Wevers M Natural Killer Cell Infiltration Is Discriminative for Antibody-Mediated Rejection and Predicts Outcome after Kidney Transplantation. Kidney Int (2019) 95:188–98. 10.1016/j.kint.2018.08.027 30396694

[90] Sis B Jhangri GS Bunnag S Allanach K Kaplan B Halloran PF. Endothelial Gene Expression in Kidney Transplants with Alloantibody Indicates Antibody-Mediated Damage Despite Lack of C4d Staining. Am J Transplant (2009) 9:2312–23. 10.1111/j.1600-6143.2009.02761.x 19681822

[91] Dominy KM Roufosse C De Kort H Willicombe M Brookes P Behmoaras JV Use of Quantitative Real Time Polymerase Chain Reaction to Assess Gene Transcripts Associated with Antibody-Mediated Rejection of Kidney Transplants. Transplantation (2015) 99:1981–8. 10.1097/tp.0000000000000621 25675206

[92] Halloran PF Pereira AB Chang J Matas A Picton M De Freitas D Microarray Diagnosis of Antibody-Mediated Rejection in Kidney Transplant Biopsies: an International Prospective Study (INTERCOM). Am J Transplant (2013) 13:2865–74. 10.1111/ajt.12465 24119109

[93] Reeve J Böhmig GA Eskandary F Einecke G Lefaucheur C Loupy A Assessing Rejection-Related Disease in Kidney Transplant Biopsies Based on Archetypal Analysis of Molecular Phenotypes. JCI Insight (2017) 2:e94197. 10.1172/jci.insight.94197 PMC5470931 28614805

[94] Lerut E Naesens M Kuypers DR Vanrenterghem Y Van Damme B. Subclinical Peritubular Capillaritis at 3 Months Is Associated with Chronic Rejection at 1 Year. Transplantation (2007) 83:1416–22. 10.1097/01.tp.0000266676.10550.70 17565313

[95] Haas M Montgomery RA Segev DL Rahman MH Racusen LC Bagnasco SM Subclinical Acute Antibody-Mediated Rejection in Positive Crossmatch Renal Allografts. Am J Transplant (2007) 7:576–85. 10.1111/j.1600-6143.2006.01657.x 17229067

[96] Loupy A Hill GS Suberbielle C Charron D Anglicheau D Zuber J Significance of C4d Banff Scores in Early Protocol Biopsies of Kidney Transplant Recipients with Preformed Donor-specific Antibodies (DSA). Am J Transplant (2011) 11:56–65. 10.1111/j.1600-6143.2010.03364.x 21199348

[97] Cosio FG El Ters M Cornell LD Schinstock CA Stegall MD. Changing Kidney Allograft Histology Early Posttransplant: Prognostic Implications of 1‐Year Protocol Biopsies. Am J Transplant (2016) 16:194–203. 10.1111/ajt.13423 26274817

[98] Papadimitriou JC Drachenberg CB Ramos E Kukuruga D Klassen DK Ugarte R Antibody-Mediated Allograft Rejection. Transplantation (2013) 95:128–36. 10.1097/tp.0b013e3182777f28 23222897

[99] Tsuji T Yanai M Itami H Ishii Y Akimoto M Fukuzawa N Microvascular Inflammation in Early Protocol Biopsies of Renal Allografts in Cases of Chronic Active Antibody-Mediated Rejection. Nephrology (2015) 20(Suppl. 2):26–30. 10.1111/nep.12450 26031582

[100] Parajuli S Joachim E Alagusundaramoorthy S Blazel J Aziz F Garg N Subclinical Antibody-Mediated Rejection after Kidney Transplantation: Treatment Outcomes. Transplantation (2019) 103:1722–9. 10.1097/tp.0000000000002566 30507740

[101] Wiebe C Rush DN Gibson IW Pochinco D Birk PE Goldberg A Evidence for the Alloimmune Basis and Prognostic Significance of Borderline T Cell-Mediated Rejection. Am J Transplant (2020) 20:2499–508. 10.1111/ajt.15860 32185878 PMC7496654

[102] Burton SA Amir N Asbury A Lange A Hardinger KL. Treatment of Antibody-Mediated Rejection in Renal Transplant Patients: a Clinical Practice Survey. Clin Transplant (2015) 29:118–23. 10.1111/ctr.12491 25430052

